# Recurrent vincristine‐associated fever in a child with Wilms tumor

**DOI:** 10.1002/cnr2.1673

**Published:** 2022-07-05

**Authors:** Eman T. Al‐Antary, Sarah Ramiz

**Affiliations:** ^1^ Division of Pediatric Hematology‐Oncology, Department of Pediatrics Children's Hospital of Michigan Detroit Michigan USA; ^2^ College of Medicine Central Michigan University Saginaw Michigan USA

**Keywords:** acetaminophen, dexamethasone, fever, pediatric, vincristine

## Abstract

**Background:**

Fever is a common complaint among children with an underlying oncologic diagnosis, especially during chemotherapy courses and periods of neutropenia. Chemotherapy‐induced fever is well described in relation to specific chemotherapy agents. However, fever induced by vincristine (VCR) has only been rarely reported.

**Case:**

We describe a case of a 5‐year‐old female with stage III Wilms tumor who had recurrent VCR‐associated fever that was controlled with prophylactic dexamethasone and acetaminophen.

**Conclusion:**

In patients developing recurrent fever following chemotherapy with VCR, febrile allergic reaction and prophylactic treatment should be considered after exhaustion of appropriate investigations.

## 1 INTRODUCTION

Wilms tumor (WT) or nephroblastoma is a rare tumor in general but is the most common pediatric malignancy of the kidney. Treatment of such a tumor is based on stage classification but usually composed of combination therapy, including chemotherapy (vincristine (VCR)‐based regimen), surgery, and radiation, which led to significantly improved outcomes with 5‐year survival up to 90%.^1^ One of the common side effects especially during chemotherapy courses and periods of neutropenia is fever, which often necessitates presentation to the emergency department and hospitalization for evaluation of sepsis. However, the cause(s) of the fever is (are) not usually clear.^2^ Chemotherapy‐induced fever is well described in relation to specific chemotherapy agents. However, fever induced by VCR has only been rarely reported.^3^, ^4^ The most commonly reported VCR side effect is neurotoxicity, which would lead to dose limitation,^5^ other less common side effects are syndrome of inappropriate antidiuretic hormone secretion,^6^, ^7^, ^8^, ^9^ myelosuppression, and alopecia.^10^

Chemotherapy (specifically VCR) induced fever prevention and management are not well established yet. We describe a case of a 5‐year‐old female with stage III WT who had recurrent VCR‐associated fever that was controlled with prophylactic dexamethasone and acetaminophen, which, up to our knowledge, is the first regimen to be reported in the literature leading to complete resolution of drug‐induced fever. Our case adds to the body of literature defining a rare but probable adverse event and its suggested management of a commonly used chemotherapy in both adults and pediatrics.

## 2 CASE PRESENTATION

Our patient is a 5‐year‐old female who presented to Children's Hospital of Michigan with abdominal pain and constipation. Initial laboratory tests were unremarkable except for anemia of chronic disease without hematuria. Ultrasound of the abdomen showed 10.9 × 9.6 × 10.5 cm left abdominal mass, likely renal in origin with compression of the left renal pelvis with mild obstructive left hydronephrosis. Magnet resonance image (MRI) of the abdomen showed 8.8 × 8.4 × 9.8 cm left renal mass, with rupture of the tumor capsule and extensive left perinephric tumor infiltration with moderate to large ascites. The mass appears to arise in the region of the renal hilum and splays the kidney around its margins resulting in obstructive hydronephrosis and delayed excretion by the left kidney. Due to the extent of the tumor, already ruptured capsule, and close proximity to the vessels, she underwent a biopsy instead of total resection. Pathology revealed WT with favorable histology. She was finally diagnosed with stage III WT. She was initiated on treatment per AREN0532 protocol arm DD4A chemotherapy (VCR 1.5–2 mg/m^2^/dose IV, Dactinomycin 0.045 mg/kg/dose IV, Doxorubicin 30–45 mg/m^2^/dose IV).

She repeatedly developed fever within 24 h (12–24 h) after receiving chemotherapy from week 1 to 4 of treatment. Interestingly, the fever was noted to persistently occur in the same pattern and time range whenever VCR was administered as a single agent or in combination with other agents. As per institutional guidelines, the patient was hospitalized for evaluation of underlying sepsis with each fever the first 4 weeks consecutively. Fevers were high grade and lasted up to 48 h. During fever episodes, her absolute neutrophil count nadir in only one encounter was 700 cells/mm^3^ (otherwise the absolute neutrophil count was ranging between 1200 and 5000 cells/mm^3^), and there were no other associated clinical symptoms, and laboratory tests remained stable with normal total and differential white blood cell count, and negative infectious work up, including imaging, viral testing, and urine and blood cultures.

Anticipating an underlying allergic reaction leading to drug‐induced fever as a probable adverse event of VCR we subsequently prophylactically administered dexamethasone 3 mg/m^2^/dose PO along with acetaminophen 15 mg/kg/dose PO one dose each prior and 12 h after each VCR administration till end of therapy (total of two doses of each medication given weekly from week 5 to week 25). Thereafter, the chemotherapy course including weekly VCR was uneventful and there were no reported recurring fevers. Moreover, the patient had no noted or reported toxicity from the prophylactic regimen. Of note, we elected not to use ibuprofen given the nephrotoxic risk as the patient has one kidney following her disease surgical control.

Besides receiving chemotherapy, the patient underwent tumor resection and left nephrectomy with a good response to chemotherapy with <10% viability. She also received total abdominal radiation of 10.5 Gy. Currently, the patient is 2 years off therapy in remission, growing and developing up to age with good quality of life.

## 3 DISCUSSION

In this report, we describe a case of a 5‐year‐old female with stage III WT who had recurrent VCR‐associated fever who was successfully managed with a prophylaxis regimen composed of two doses of dexamethasone and acetaminophen given weekly (from week 5 to 25 of chemotherapy), which is, up to our knowledge, is the first to be reported in the literature.

Fever during chemotherapy for cancer has been reported to occur in around a third of the patients, secondary to different causes, of which 24% are chemotherapy‐induced, most commonly occurring in the first few days post‐treatment, in contrast to an infection‐induced fever that peaks during the neutropenia or after the first week of treatment.^11^ Our patient was developing fevers within the first 24 h following receiving chemotherapy, more consistent with VCR administration as a probable adverse event as per WHO‐UMC Criteria for causality assessment.^12^

Many antineoplastic agents like bleomycin, etoposide, cisplatin, daunorubicin, hydroxyurea, vincristine, cladribine, gemcitabine, and 6‐mercaptopurine, among others, and monoclonal antibodies can induce fever.^13^ The pathophysiology behind this phenomenon is not well established yet, especially for chemotherapy like VCR. Imai et al suggested that an allergic response to VCR might be involved when investigating recurrent fever in a 2‐year‐old patient with rhabdomyosarcoma after receiving VCR including chemotherapy.^3^ Leukocyte migration testing was performed which showed that the migration index with VCR added to the patient's serum was significantly higher compared to normal controls. These findings indicated the possibility of an underlying delayed cell‐mediated hypersensitivity to VCR.

Ishii et al reported that more than two VCR‐induced fever episodes were identified in nine of 31 children with leukemia or lymphoma undergoing maintenance chemotherapy. Similarly, they reported that the duration of fever was shortened but not completely prevented with corticosteroids suggesting the possibility of an allergic reaction mechanism behind it.^4^ This is supporting our management as we used dexamethasone in addition to acetaminophen before and the night after administering VCR containing chemotherapy regimen, but in our case, that regimen resulted in complete prevention of recurrence of VCR‐associated fever. Given the short course of this prophylactic regimen with each VCR dose, our patient did not develop clinical toxicities. Indicating that this suggested regimen is effective and safe in such a clinical scenario. Another anti‐inflammatory medication that might be considered in such a situation is a non‐steroidal anti‐inflammatory drug like ibuprofen as long as the suspected risk of bleeding or nephrotoxicity in these patients is low.

In conclusion, VCR‐associated fever appears to be an interesting but rare phenomenon, given the wide application of VCR in adult and pediatric chemotherapy protocols and the scarcity of reports in the literature presenting such a side effect. Therefore, we hope to support the previous reports with our individual experience. We conclude that in patients developing recurrent fever following chemotherapy with VCR, febrile allergic reaction and prophylactic treatment should be considered after exhaustion of appropriate investigations and other differential causes, which will prevent unnecessary admissions and antibiotic use.

## AUTHOR CONTRIBUTIONS

**Eman Al‐Antary:** Conceptualization (lead); data curation (lead); methodology (lead); writing – original draft (lead); writing – review and editing (lead). **Sarah Ramiz:** Supervision (lead); writing – original draft (supporting); writing – review and editing (supporting).

## CONFLICT OF INTEREST

The authors have stated explicitly that there are no conflicts of interest in connection with this article.

## ETHICS STATEMENT

This is a case report summarizing the patient's clinical course. The Children's Hospital of Michigan Research Ethics Committee has confirmed that no ethical approval is required.

## Data Availability

The patient data summarized in the current report are available from the corresponding author on reasonable request.
